# Assessment of the Value of Tumor Variation Profiling Perceived by Patients With Cancer

**DOI:** 10.1001/jamanetworkopen.2020.4721

**Published:** 2020-05-14

**Authors:** Phyllis Butow, Grace Davies, Christine E. Napier, Timothy Schlub, Megan C. Best, Nicole Bartley, Ilona Juraskova, Bettina Meiser, Mandy L. Ballinger, Barbara Biesecker, David Goldstein, David M. Thomas

**Affiliations:** 1 Psycho-Oncology Co-Operative Research Group, Faculty of Science, The University of Sydney School of Psychology, Sydney, New South Wales, Australia; 2 Centre for Medical Psychology and Evidence-Based Decision-Making, The University of Sydney School of Psychology, Sydney, New South Wales, Australia; 3 Cancer Division, Garvan Institute of Medical Research, Darlinghurst, New South Wales, Australia; 4 Sydney School of Public Health, Faculty of Medicine and Health, The University of Sydney, Sydney, New South Wales, Australia; 5 Psychosocial Research Group, Prince of Wales Clinical School, University of New South Wales, Kensington, New South Wales, Australia; 6 RTI International, Washington, DC; 7 Department of Medical Oncology, Prince of Wales Hospital, Randwick, New South Wales, Australia

## Abstract

**Importance:**

Use of tumor molecular profiling (MP) is entering routine clinical practice; however, little is known about how much and why patients value MP.

**Objective:**

To examine the perceived value of MP to patients with advanced cancer and factors associated with perceived value.

**Design, Setting, and Participants:**

A cross-sectional survey that included willingness-to-pay trade-off scenarios was administered to participants after consent and before MP. A total of 777 participants (94% response rate) were recruited from the Molecular Screening and Therapeutics Program. Eligible patients had advanced solid cancers of any histologic type, were receiving or had completed their last line of effective therapy, had an Eastern Cooperative Oncology Group Performance Status 0 to 3, and had sufficient accessible tissue for MP. The participants were recruited between October 24, 2017, and March 12, 2019, and data analysis was conducted from March 13 to April 14, 2019.

**Main Outcomes and Measures:**

Willingness to pay for MP was assessed via hypothetical trade-off scenarios varying in the actionable return rate (1%, 20%, or 40%) and cost (A$0, A$300 [US$210], A$1000 [US $700], A$3000 [US $2100], or A$10 000 [US $7000]). Ordinal regressions were used to explore factors associated with willingness to have and pay for MP.

**Results:**

Of 777 participants (405 women [52%]; mean [SD] age, 55.47 [14.26] years), 689 patients (89%) would have MP for as little as a 1% actionable return rate. Fifty-six patients (7%) would require at least a 20% return rate and 11 patients (1%) would require at least a 40% return rate. Fifteen patients (2%) consistently chose not to have the test; 6 participants (0.8%) had missing values on this item. Participants were willing to pay a median of A$1000 if the actionable return rate was 1% and A$3000 for an actionable return rate of 20% to 40%. Of 762 individuals who agreed to testing, 482 patients (64%) were consistently unwilling to pay A$10 000, regardless of the actionable return rate. Patients born in Australia or New Zealand were more likely to want MP (eg, participants born in South Asia had an ordered odds for the tipping point of 7.74 [95% CI, 1.67-36.05; *P* = .009] times higher than Australian- and/or New Zealand–born participants). Patients born in Australia or New Zealand were also more willing to pay A$1000 or A$3000 (eg, participants born in Western Europe had an ordered odds for the tipping point for paying A$1000 of 1.74 [95% CI, 1.01-3.00; *P* = .048] times higher than Australian- and/or New Zealand–born participants). People with a medical- or science-related occupation and with more negative attitudes toward uncertainty were more likely to pay A$10 000 (eg, A$10 000 tipping point–ordered odds of participants with a medical- or science-related occupation was 0.49 [95% CI, 0.7-0.87; *P* = .02] times that of participants without a medical- or science-related occupation).

**Conclusions and Relevance:**

This study found apparent high interest in but lower willingness to pay for MP among patients with advanced cancer. Ability to pay may limit access to MP. Ongoing societal debate is required to establish the value of MP and whether subsidization is needed to ensure equity of access.

## Key Points

**Question:**

What value do patients with advanced cancer place on tumor variation profiling?

**Findings:**

In this survey study of 777 patients with advanced cancer, 689 individuals (89%) indicated that they would have tumor molecular profiling for as little as a 1% actionable return rate, but fewer were willing to pay for it. Country of birth, medical- or science-related occupation, attitude toward uncertainty, and perceived importance of tumor molecular profiling were associated with willingness to pay.

**Meaning:**

Ability to pay may limit access to tumor molecular profiling; ongoing societal debate is required to establish the value of tumor molecular profiling and whether subsidization is necessary to ensure equity of access.

## Teaser

This survey study examines the acceptance of and willingness to pay for molecular profiling of tumors by patients with advanced cancer.

## Introduction

Oncology is leading the clinical integration of genomic technologies into medicine with an increasing focus on defining cancer by its molecular signatures as opposed to its histologic characteristics.^1^ In the advanced cancer setting, this process generally involves somatic molecular profiling (MP) via panel testing of solid tumors to identify cancer-specific gene variations to guide targeted treatment. While the potential benefit of MP is high, challenges to its routine use exist, in particular, assessment of the utility of returned results.

Molecular profiling of tumors can identify variants that (1) guide treatment through a standard regimen or clinical trial (clinically actionable), (2) cannot guide treatment (nonactionable), (3) have uncertain therapeutic potential, and (4) occasionally have a germline origin and, therefore, have relevance to the patient’s family. Currently, the likelihood of finding a clinically actionable variant and obtaining improved outcomes is unknown and evolving rapidly. In a study of patients with advanced breast cancer,^2^ of 297 participants with biopsies for genomic analysis, personalized treatment was available for 55 individuals (18.5%). Of 43 patients treated with 16 different targeted regimens, 4 patients had objective responses and 9 patients had stable disease. Similarly, in a population-based study of 875 patients with advanced non–small cell lung cancer who received broad-based genomic sequencing,^3^ less than 5% of the participants were given nonapproved targeted treatments based on sequencing results. These treatments were not independently associated with higher 12-month survival rates. However, a more recent study of biomarker-driven therapy in the I-PREDICT trial^4^ resulted in matched therapy in 49% of patients screened. Targeting of molecular alterations was correlated with significantly improved disease control rates, as well as longer progression-free and survival rates. As evidence advances quickly, the perceived value of MP has become a topic of heightened debate.

While there a number of stakeholders whose perspectives might inform this debate,^5^ the patient’s perspective is a highly relevant factor. In considering the value of MP, patients need to consider financial and other costs against perceived clinical benefits. Recently, the cost of MP in Australia has fallen from A$6000 (US $4200) to under A$1000 (US $700) (David M. Thomas, PhD, email communication, June 18, 2019). Nevertheless, the lower cost still represents a significant investment for the health system and potential financial hardship for patients. Another perceived cost may be time spent waiting for results^6^ during which living with uncertainty can cause uneasiness and anxiety.^7^ Patients may have additional concerns related to the privacy, security, and ethics of testing.^8^

Psychological theories suggest that individual differences may be factors in how patients evaluate clinical benefits vs costs. Protection motivation theory,^9^ for example, would suggest that individuals will value MP more and place less weight on costs if they perceive cancer to be a significant threat, feel vulnerable to cancer progression, are confident that MP-guided treatment will be effective, and are confident that they will cope with MP-guided treatment. Protection motivation theory has been successful in estimating health- and safety-related intentions and behaviors in a variety of contexts.^9^

Few studies have empirically assessed patients’ views on MP. One useful method for establishing value is the willingness-to pay approach. Willingness to pay is a measure of strength of preference for or value given to a commodity such as a medical treatment.^10^ Cuffe et al^11^ used this approach in examining the value of pharmacogenomic testing to identify the likelihood of experiencing significant chemotherapy-related toxic effects to patients with metastatic cancers. The investigators used hypothetical time, efficacy, and toxic effect trade-offs and willingness-to-pay scenarios. In 121 participants, 97.4% wanted pharmacogenomic testing that could detect the risk of severe toxic effects and were willing to accept a median cost of A$1000 (range, A$0-A$10 000) and a waiting time for results of 14 days (range, 1-90 days).

The present study aimed to examine the perceived value of MP by exploring willingness to have and pay for MP and associated factors in a cohort of patients who have consented to MP as part of a genomic study. On the basis of the literature^12^ and the protection motivation theory, we hypothesized that participants would highly value MP and that those who have children, a medical or other scientific background, a feeling of vulnerability to cancer progression, good knowledge of genomics, high self-efficacy in coping with MP-guided treatment, a negative attitude toward uncertainty, and high fear of cancer progression may value MP more than those without these characteristics.

## Methods

### Participants

This project is a substudy of the Molecular Screening and Therapeutics (MoST) Program,^13^ an ongoing study at the Garvan Institute of Medical Research in Sydney, Australia. The MoST Program is enrolling adult participants with pathologically confirmed advanced or metastatic solid cancers of any histologic type, either during or after their last line of effective therapy; participants have an Eastern Cooperative Oncology Group (ECOG) Performance Status of 0 to 3 (ie, 0, no restrictions; 1, patient is restricted in physically strenuous activity but ambulatory and able to carry out light or sedentary work; 2, patient is ambulatory more than 50% of waking hours and capable of all self-care but unable to carry out any work activities; 3, patient is capable of only limited self-care, confined to bed or chair more than 50% of waking hours)^14^ and sufficient accessible tissue for MP. If actionable findings are reported, participants may be enrolled in a targeted therapeutic trial or treatment. Recruitment for the MoST Program is through oncologist referral.

The Psychosocial Issues in Genomics in Oncology (PiGeOn) Project is a longitudinal, mixed-methods, psychosocial substudy of the MoST Program that aims to examine the psychosocial, behavioral, and ethical aspects of MP.^15^ Exclusion criteria are inability to adhere to study requirements, including completing assessments, or to provide written informed consent. Participants give written informed consent to participate in the PiGeOn Project while giving consent to participate in the MoST Program; no financial compensation was provided. The PiGeOn Project was approved by the St Vincent’s Hospital Human Research Ethics Committee, Sydney, Australia. This study followed the American Association for Public Opinion Research (AAPOR) reporting guideline.

PiGeOn Project participants complete a questionnaire at baseline (within 2 weeks of consenting to MP but before receipt of results), 2 to 3 months post baseline (shortly after receiving MP results), and 2 to 3 months later. This article reports results only from the baseline survey.

### Measures

A hypothetical trade-off scenario using a contingent valuation willingness-to-pay approach^12^ based on previous studies^11^ was used to measure the degree to which participants value MP. Two approaches to willingness to pay have primarily been used in the literature: contingent valuation and discrete choice experiments. These approaches both have pros and cons and produce slightly different results. We chose contingent valuation because it is closer to the real choices consumers make.^12^ Patients were presented with 3 different scenarios with varying likelihoods that MP would find an actionable variant (1%, 20%, or 40%): If MP found a gene variant to guide treatment in about 1 in 100 people (1%), would you have the test? If MP found a gene variant to guide treatment in about 20 in 100 people (20%), would you have the test? If MP found a gene variant to guide treatment in about 40 in 100 people (40%), would you have the test? For each actionable result return rate, participants indicated whether they would have the test (yes/no) and, if yes, the highest amount they would pay (A$0, A$300 [US $210], A$1000 [US $700], A$3000 [US $2100], or A$10 000 [US $7000]). It was emphasized to participants that the choices were hypothetical, as they would be provided with MP at no cost within the MoST Program.

Demographic and psychological variables were collected. These measures included sex, age, educational level, cultural and linguistic background, socioeconomic status, country of birth, urban vs rural status, previous visit to a family cancer clinic, occupation, and parental status.

A 2-item measure adapted from Hay et al^16^ assessed perceived importance of genetic information to the participant, using a Likert-type scale. Higher scores indicate greater importance. Participants indicate their perceived likelihood of cancer progression on a visual analog scale from 0% (no chance of progression) to 100% (will definitely progress) adapted from a previous study.^17^

An 8-item, multiple-choice, purposively developed measure assessed knowledge of the purpose of MP, likely frequency of informative results, cancers in which informative results are more likely to be found, and availability of tailored treatment options. High scores (summed) indicate greater knowledge. The 3-item Concerns About Recurrence Questionnaire^18^ was adapted to measure fear of cancer progression. High scores indicate greater fear. The 7-item Attitude Toward Uncertainty scale^19^ measures uncertainty in the context of medical testing. Higher scores indicate negative attitudes toward uncertainty.

Four items adapted from Rosenberg et al^20^ assessed perceived ability to cope if actionable or nonactionable results are found. High scores indicate greater perceived ability to cope. The 6-item Satisfaction With Decision scale^21^ measures satisfaction with the person’s decision to have MP. High scores indicate greater satisfaction.

### Statistical Analysis

Descriptive statistics were used to summarize responses. Four tipping-point scores were computed for each participant using the hypothetical trade-off scenarios. The first tipping-point score was determined using the minimum actionable result return rate (1%, 20%, and 40%) required for patients to say yes to having the MP test. Patient responses formed 4 ordered categories: those who would have the test with a 1% likelihood, a 20% likelihood, or a 40% likelihood of finding an actionable variant, and those who consistently would not have the test. Hence, if a patient said no to the first 2 scenarios (1% and 20%) and then yes at 40%, they would receive a tipping-point score of 40, placing them in the third category. Three monetary tipping points were computed based on the actionable result return rate required for patients to spend A$1000, A$3000, and A$10 000 for the test. Lower tipping-point categories represent greater value placed on MP tests.

Pearson correlation coefficients were used to explore associations between variables of interest. Ordinal regressions were conducted on the trade-off categories, that is, participants’ tipping points for when they would have the test and for when they would pay A$1000, A$3000, or A$10 000 for MP as the outcome variables. All demographic and psychological factors measured were included as independent variables in these analyses. Listwise deletion was chosen to manage missing data as this process is known to produce unbiased estimates and conservative results.^22^ Wald tests were used to examine whether explanatory variables added significant explanatory power to the model.

The sample size and power calculation for this study are reported in the protocol.^15^ Owing to the large number of *P* values reported in this article, readers should be aware that the global type I error rates would be larger than expected by choosing a fixed significance-level cutoff. We therefore recommend that *P* values be treated as an indicator for the level of evidence for a difference in any comparison rather than as a binary outcome of significant and not significant. The American Statistical Association statement on *P* values provides further guidance on interpreting statistical results including *P* values.^23^ Data analysis was conducted using SPSS Statistics, version 25 (IBM Corp).

## Results

In this cross-sectional survey, using willingness-to-pay trade-off scenarios, 777 participants were recruited between October 24, 2017, and March 12, 2019, and completed the baseline questionnaire as part of the PiGeOn Project study (a 94% response rate). Data were analyzed between March 13 and April 14, 2019. Of the 52 nonresponders, 6 died shortly after consenting and the remainder chose not to participate, citing lack of interest, capacity, or time.

Participants had a mean (SD) age of 55.47 (14.26) years (interquartile range, 19-90 years), with 372 men (48%) and 405 women (52%) (Table 1). Nearly half (345 [44%]) had a university education, 59 patients (8%) had a medical/scientific background, and 86 patients (11%) had previously visited a family cancer clinic. There were 247 participants (32%) not born in Australia or New Zealand, with 184 individuals (24%) speaking a language other than English at home. Seventy-five patients (10%) lived in rural/remote areas of Australia and 574 patients (74%) had children. There were few missing data, the highest being 10 responses for any variable.

**Table 1.** Demographic Characteristics

Characteristic	No. (%)
No.	777 (94)
Sex	
Female	405 (52)
Male	372 (48)
Age, y	
Mean (SD)	55.47 (14.26)
Median (IQR) [range]	58 (20) [19-90]
Highest level of education completed	
Primary school (some or all)	9 (1)
Secondary school	
Year 7 or 8	23 (3)
Year 9 or 10	124 (16)
Year 11 or 12	133 (17)
Vocational training	137 (18)
University	
Undergraduate	177 (23)
Postgraduate	168 (22)
Missing	6 (1)
CALD (speaks a language other than English)	184 (24)
SES, mean (SD) [range]	6.78 (2.86) [1-10]
Country of birth	
East Asia and Pacific	65 (8)
South Asia	14 (2)
Sub-Saharan Africa	10 (1)
Middle East and Northern Africa	27 (3)
Central and South America	11 (1)
North America	7 (1)
Europe	
Eastern	11 (1)
Southern	29 (4)
Northern	7 (1)
Western	66 (8)
Australia and New Zealand	530 (68)
ARIA	
Urban	702 (90)
Remote/rural	75 (10)
Visited a family cancer clinic	86 (11)
Medical- or science-related occupation	59 (8)
Parent	574 (74)
No. of children, range	1-8
Primary site of cancer	
Bone and soft tissue	146 (19)
Brain	93 (12)
Colorectal	71 (9)
Pancreas	69 (9)
Breast	44 (6)
Uterus	44 (6)
Ovary	37 (5)
Unknown primary	27 (3)
Lung	24 (3)
Prostate	20 (3)
Other	202 (26)
ECOG performance status^a^	
0	393 (51)
1	345 (44)
2	31 (4)
3	2 (0.3)
Satisfaction with decision to have MP, mean (SD) [range]^b^	4.40 (0.75) [1-5]
Self-efficacy, mean (SD) [range]^c^	4.29 (0.73) [1-5]
Attitude toward uncertainty, mean (SD) [range]^d^	4.31 (0.56) [1.5-5]
Perceived susceptibility, mean (SD) [range]^e^	65.07 (27.32) [0-100]
Concerns of cancer recurrence, mean (SD) [range]^f^	58.68 (26.71) [0-100]
Perceived importance, mean (SD) [range]^g^	4.73 (0.60) [1-5]
Knowledge, mean (SD) [range]^h^	0.43 (0.20) [0.00-0.88]

Abbreviations: ARIA, Accessibility and Remoteness Index of Australia; CALD, culturally and linguistically diverse; ECOG, Eastern Cooperative Oncology Group; MP, molecular profiling; SES, socioeconomic status.

^a^ ECOG assesses the patients’ daily living abilities to guide appropriate treatment and prognosis: 0, no restrictions; 1, patient is restricted in physically strenuous activity but ambulatory and able to carry out light or sedentary work; 2, patient is ambulatory more than 50% of waking hours and capable of all self-care but unable to carry out any work activities; 3, patient is capable of only limited self-care, confined to bed or chair more than 50% of waking hours.

^b^ Six-item Satisfaction With Decision scale; higher scores indicate greater satisfaction.

^c^ Higher scores indicate greater perceived ability to cope.

^d^ Seven-item Attitude Toward Uncertainty scale; higher scores indicate negative attitudes toward uncertainty.

^e^ Perceived likelihood of cancer progression on a visual analog scale from 0% (no chance of progression) to 100% (will definitely progress).

^f^ Concerns About Recurrence Questionnaire; higher scores indicate greater fear.

^g^ Two-item measure using Likert-type scale; higher scores indicate greater importance.

^h^ Eight-item, multiple-choice measure; scores are summed and divided by the total number of items; higher scores indicate greater knowledge about MP.

### Tipping Points

Responses to scenarios for receiving MP and willingness to pay are shown in Table 2, and tipping points are summarized in Table 3. Six hundred eighty-nine participants (89%) would have MP with only a 1% likelihood of finding an actionable variant. An additional 56 participants (7%) would require at least a 20% return rate and 11 (1%) participants would require at least a 40% return rate to have MP. Fifteen patients (2%) consistently chose not to have the test despite the increasing likelihood for detection, while 6 participants (0.8%) showed irregularly patterned responses.

**Table 2.** Willingness to Have and Pay for Variation Profiling

Response^a^	No. (%)
**If MP found a gene variant to guide treatment in about 1 in 100 people (1%), would you have the test?**	
Yes	689 (89)
No	82 (11)
Missing	6 (0.8)
Highest amount you would be prepared to pay, A$	
0	69 (10)
300	166 (24)
1000	150 (22)
3000	119 (17)
10 000	174 (25)
Missing	11 (2)
**If MP found a gene variant to guide treatment in about 20 in 100 people (20%), would you have the test?**	
Yes	744 (96)
No	28 (4)
Missing	5 (0.6)
Highest amount you would be prepared to pay, A$	
0	65 (9)
300	144 (19)
1000	154 (21)
3000	166 (22)
10 000	202 (27)
Missing	13 (2)
**If MP found a gene variant to guide treatment in about 40 in 100 people (40%), would you have the test?**	
Yes	756 (97)
No	16 (2)
Missing	5 (0.6)
Highest amount you would be prepared to pay, A$	
0	66 (9)
300	121 (16)
1000	139 (18)
3000	165 (22)
10 000	252 (33)
Missing	13 (2)

Abbreviation: MP, molecular profiling.

^a^ Monetary results shown as Australian dollars: A$300 = US $210, A$1000 = US $700, A$3000 = US $2100, A$10 000 = US $7000.

**Table 3.** Tipping-Point Scores for Having the Test and Payment Levels

Tipping points^a^	Participants who had this tipping point, No. (%)
**To have the test**^b^	
Variation rate	
1%	689 (89)
20%	56 (7)
40%	11 (1)
Would invariably not take the test	15 (2)
Missing/irregular responses	6 (0.8)
**To pay at least A$1000**^b^	
Variation rate	
1%	434 (57)
20%	87 (11)
40%	34 (4)
Consistently would not pay A$1000	180 (24)
Missing/irregular responses	27 (3)
**To pay at least A$3000**^b^	
Variation rate	
1%	286 (38)
20%	80 (10)
40%	51 (7)
Consistently would not pay A$3000	320 (42)
Missing/irregular responses	25 (3)
**To pay A$10 000**^**b**^	
Variation rate	
1%	166 (22)
20%	36 (5)
40%	52 (7)
Consistently would not pay A$10 000	482 (64)
Missing/irregular responses	26 (3)

^a^ Monetary results shown as Australian dollars: A$300 = US $210, A$1000 = US $700, A$3000 = US $2100, A$10 000 = US $7000.

^b^ Of 762 who said yes to testing.

For participants who would have the test, the median amount they were prepared to pay if the actionable return rate was 1% was A$1000, and if the actionable return rate was 20% to 40%, they were willing to pay A$3000 (Figure). Of 762 patients willing to undergo testing, 555 individuals (73%) would pay at least A$1000 for MP, with 434 patients (57%) willing to do so for a 1% chance of returning a clinically actionable result. Only 417 participants (55%) were willing to pay A$3000 for MP, of whom 286 patients (38%) were willing to do so for a 1% return of a clinically actionable result. Only 254 patients (33%) were willing to pay A$10 000, of whom 166 of 254 individuals (22%) were willing to do so for a 1% return rate. In all cases, the increase in return rate from 1% to 20% or 40% had a minor association with the decision to pay for the test.

**Figure.**
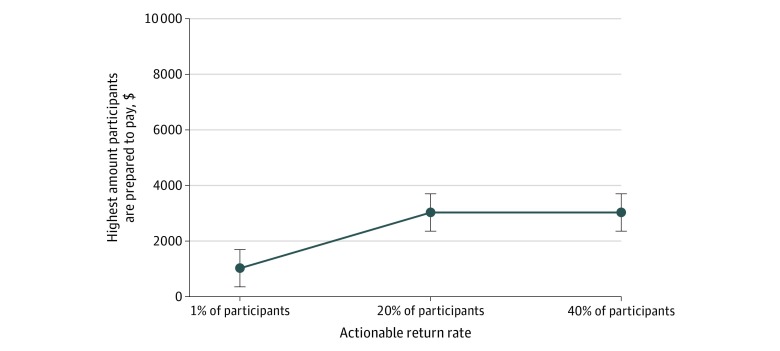
Median Amount Participants Are Willing to Pay as a Function of Actionable Return Rate Results shown in Australian dollars: A$1000 [US $700], A$3000 [US $2100]. Error bars indicate 95% CI.

### Factors Associated With the Tipping Point 

Country of birth and perceived importance of MP were associated with the tipping point for having MP (eFigure in the Supplement; Table 4). Participants born in South Asia had an ordered odds for the tipping point of 7.74 (95% CI, 1.67-36.05; *P* = .009) times higher than Australian- or New Zealand–born participants. Similarly, compared with participants born in Australia or New Zealand, the ordered odds for the tipping point for participants born in the Middle East or Northern Africa were 5.56 (95% CI, 1.75-17.62; *P* = .004) times greater; for those born in Central or South America, the ordered odds increased by a factor of 6.01 (95% CI, 1.21-29.90; *P* = .03); and for participants born in Western Europe, the ordered odds increased by a factor of 2.40 (95% CI, 1.06-5.45; *P* = .04). Thus, fewer participants born in these regions chose to have the test than participants born in Australia or New Zealand when there was a low likelihood of finding a result.

**Table 4.** Summary of Regression Analyses for Variables Associated With Tipping-Point Scores

Independent variable	Ordinal regression for tipping point							
	Paying for having the test		Paying A$1000		Paying A$3000		Paying A$10 000	
	Odds ratio (95% CI)	*P* value	Odds ratio (95% CI)	*P* value	Odds ratio (95% CI)	*P* value	Odds ratio (95% CI)	P value
Sex								
Female	0.71 (0.41-1.23)	.22	1.04 (0.76-1.43)	.81	1.00 (0.74-1.36)	.98	1.08 (0.77-1.51)	.66
Male	1 [Reference]		1 [Reference]		1 [Reference]		1 [Reference]	
Age	1.00 (0.98-1.02)	.93	0.99 (0.98-1.01)	.34	0.99 (0.98-1.00)	.11	1.00 (0.99-1.01)	.91
Educational level	1.05 (0.87-1.27)	.61	0.94 (0.84-1.05)	.27	0.90 (0.81-1.00)	.06	0.93 (0.82-1.05)	.23
CALD								
Yes	0.74 (0.33-1.66)	.46	0.96 (0.59-1.56)	.85	0.95 (0.59-1.52)	.83	0.98 (0.58-1.64)	.93
No	1 [Reference]		1 [Reference]		1 [Reference]		1 [Reference]	
SES	0.98 (0.89-1.08)	.67	0.97 (0.91-1.03)	.35	0.98 (0.93-1.04)	.57	1.00 (0.94-1.07)	.91
Country of birth								
East Asia	1.35 (0.43-4.29)	.61	1.90 (0.99-3.62)	.05	2.13 (1.10-4.10)	.02	1.45 (0.69-3.01)	.32
South Asia	7.74 (1.67-36.05)	.009	2.93 (0.85-10.14)	.09	2.63 (0.72-9.66)	.14	1.85 (0.45-7.63)	.40
Sub-Saharan Africa	1.51 (0.17-13.78)	.72	1.78 (0.53-6.00)	.35	1.76 (0.51-6.03)	.37	1.53 (0.37-6.32)	.55
Middle East and Northern Africa	5.56 (1.75-17.62)	.004	1.76 (0.70-4.44)	.23	1.94 (0.76-4.93)	.16	1.46 (0.52-4.08)	.47
Central and South America	6.01 (1.21-29.90)	.03	1.42 (0.38-5.26)	.60	1.87 (0.53-6.59)	.33	2.30 (0.57-15.61)	.19
North America	Not computed^a^		0.68 (0.12-3.73)	.66	0.24 (0.04-1.40)	.11	0.51 (0.12-2.27)	.38
Eastern Europe	Not computed^a^		1.12 (0.27-4.60)	.88	0.90 (0.24-3.31)	.87	1.05 (0.26-4.26)	.95
Southern Europe	3.63 (0.94-14.01)	.06^b^	1.76 (0.70-4.46)	.23	1.07 (0.43-2.67)	.88	1.35 (0.46-3.92)	.59
Northern Europe	2.59 (0.25-26.98)	.43	1.48 (0.28-7.87)	.65	0.99 (0.20-5.00)	.99	0.62 (0.12-3.21)	.57
Western Europe	2.40 (1.06-5.45)	.04	1.74 (1.01-3.00)	.048	1.37 (0.80-2.34)	.25	1.34 (0.74-2.43)	.30
Australia or New Zealand	1 [Reference]		1 [Reference]		1 [Reference]		1 [Reference]	
ARIA								
Urban	2.00 (0.57-7.07)	.28	0.83 (0.48-1.45)	.52	0.81 (0.47-1.38)	.44	0.73 (0.40-1.35)	.32
Remote/rural	1 [Reference]		1 [Reference]		1 [Reference]		1 [Reference]	
Family cancer clinic								
Visited	0.64 (0.24-1.73)	.42	0.86 (0.52-1.43)	.56	1.00 (0.62-1.60)	.98	1.27 (0.75-2.16)	.38
Not visited	1 [Reference]		1 [Reference]		1 [Reference]		1 [Reference]	
Medical- or science-related occupation								
Yes	1.72 (0.66-4.52)	.27	0.92 (0.49-1.74)	.80	0.75 (0.42-1.34)	.33	0.49 (0.27-0.87)	.02
No	1 [Reference]		1 [Reference]		1 [Reference]		1 [Reference]	
Parental status								
Yes, has children	1.05 (0.50-2.21)	.89	1.10 (0.72-1.68)	.67	1.14 (0.76-1.72)	.52	0.79 (0.51-1.25)	.32
No	1 [Reference]		1 [Reference]		1 [Reference]		1 [Reference]	
Satisfaction with decision for MP	0.88 (0.60-1.29)	.52	1.14 (0.87-1.50)	.35	1.15 (0.89-1.48)	.29	1.19 (0.91-1.55)	.20
Self-efficacy	0.89 (0.57-1.38)	.59	0.77 (0.57-1.04)	.09	0.78 (0.58-1.04)	.09	0.91 (0.66-1.26)	.57
Attitude toward uncertainty	0.66 (0.38-1.15)	.15	0.77 (0.54-1.10)	.15	0.56 (0.40-0.80)	.001	0.47 (0.32-0.70)	<.001
Perceived susceptibility	1.00 (0.99-1.01)	.79	1.00 (0.99-1.00)	.36	1.00 (0.99-1.00)	.48	1.00 (1.00-1.01)	.46
Concerns of cancer recurrence	1.00 (0.99-1.01)	.49	1.00 (0.99-1.00)	.29	1.00 (0.99-1.00)	.34	1.00 (0.99-1.00)	.10
Perceived importance	0.64 (0.44-0.92)	.02	0.77 (0.58-1.03)	.08^b^	0.71 (0.52-0.96)	.03	0.92 (0.66-1.29)	.64
Knowledge	0.51 (0.13-1.95)	.32	0.56 (0.25-1.26)	.16	0.84 (0.38-1.82)	.65	0.66 (0.28-1.59)	.36

Abbreviations: ARIA, Accessibility and Remoteness Index of Australia; CALD, culturally and linguistically diverse; SES, socioeconomic status.

^a^ Regression coefficient unable to be computed for this variable owing to lack of variability.

For each category increase in participants’ perceived importance of MP (eg, from agree to strongly agree), the tipping point–ordered odds decreased by a factor of 0.64 (95% CI, 0.44-0.92; *P* = .02). Hence, participants who perceived MP as being of greater importance were eager to have the test even when there was a low likelihood of finding a result. There was a significant, although low, correlation (*r* = 0.13, *P* = .001) between perceived importance of MP and thinking that MP can always guide treatment decisions (response to a question on the knowledge scale), suggesting that some participants with positive attitudes toward MP may not recognize the low likelihood of finding a result.

### Tipping Point for Willingness to Pay 

Participants’ country of birth was associated with the tipping point for paying A$1000 for MP. For participants born in Western Europe, the A$1000 tipping point–ordered odds increased by a factor of 1.74 (95% CI, 1.01-3.00; *P* = .048). Hence, these participants were less willing than those born in Australia or New Zealand to pay A$1000, even with a low likelihood of finding a result.

Country of birth, attitudes toward uncertainty, and perceived importance were associated with the tipping point for paying A$3000 for MP. For participants born in East Asia, the A$3000 tipping point–ordered odds increased by a factor of 2.13 (95% CI, 1.10-4.10; *P* = .02) compared with Australian- or New Zealand–born participants. For every 1-category increase in attitudes toward uncertainty (eg, from agree to strongly agree), the A$3000 tipping point–ordered odds decreased by a factor of 0.56 (95% CI, 0.40-0.80; *P* = .001). For every 1-category increase in perceived importance, the A$3000 tipping point–ordered odds decreased by a factor of 0.71 (95% CI, 0.52-0.96; *P* = .03). Thus, compared with East Asia participants, those born in Australia or New Zealand who had negative attitudes to uncertainty or who perceived MP as being of greater importance were willing to pay A$3000 at a low result return rate.

A medical- or science-related occupation and attitudes toward uncertainty were the only factors assocaited with the tipping point for paying A$10 000 for MP. The A$10 000 tipping point–ordered odds of participants with a medical- or science-related occupation was 0.49 (95% CI, 0.27-0.87; *P* = .02) times that of those without a medical- or science-related occupation. Thus, participants with a medical- or science-related occupation were more likely to pay A$10 000 for MP even at low rates of success, placing greater value on MP.

For each category increase in attitudes toward uncertainty, the A$10 000 tipping point–ordered odds decreased by a factor of 0.47 (95% CI, 0.32-0.70; Wald χ^2^_1_ = 13.91; *P* < .001). Hence, participants with more negative attitudes toward uncertainty required a low likelihood of finding a result to pay A$10 000.

## Discussion

In this sample of 777 patients with advanced cancer who chose to receive MP as part of a large genomic study, most (89%) would choose to have MP, even if the likelihood of finding a gene variant that could guide treatment for their cancer was as low as 1%. Of the few who considered the likelihood of receiving an actionable result, relatively low actionable return rates of 20% or 40% tipped the balance.

These results complement the PiGeOn Project qualitative findings reported in 2019,^24^ which indicated that, for this group of participants who were reaching the end of their conventional cancer treatment options, any hope was better than none. As one man noted, “Well, it’s pretty simple. When you’ve got no other hope or no other opportunity and no other idea what the hell is going on, you grab every chance you can grab.”^24^ These patients saw MP as an opportunity to receive a tailored therapy that might increase their survival, and since the test was done on previously collected tumor tissue and was therefore noninvasive, to consent to it was described as a “no-brainer.”^24^ More unexpected in the present analysis were the few participants (n = 15) who chose not to undergo MP for any reason; responses were missing on 6 participants. Perhaps these patients were participating in the MoST Program for altruistic reasons and had greater faith in their oncologist to guide their care than in genomics.

More participants born in Australia or New Zealand chose to have the test when there was a low actionable return rate than participants born in other parts of the world. Similarly, these individuals were more willing to pay A$1000 or A$3000 for MP. Other studies have found that individuals of minority racial/ethnic groups or those who were immigrants had less-positive attitudes to genetic testing, feared discrimination as a result of testing, and anticipated a more negative emotional reaction to receipt of results than native-born participants,^25,26,27^ which may explain our result. We have also found higher levels of anxiety and depression in patients with cancer who were immigrants compared with native-born, as well as difficulty navigating the health system and understanding the English language,^28^ which may make genomic and treatment decisions more difficult.

Those who perceived MP as important were also more likely to choose to have MP, even with low return rates. The significant correlation between perceived importance and agreeing that MP is always helpful for guiding treatment decisions for cancer suggests that these participants may have believed there was a high likelihood of finding an actionable result, even when they were responding to a hypothetical low return rate.

Despite the strong value placed on MP among this cohort, when faced with a monetary value, significantly fewer were prepared to pay for MP, with 180 participants (24%) not willing to pay even A$1000, which is a realistic cost in Australia at the time of publication. The median amounts participants were prepared to pay was A$1000 if the actionable return rate was 1% and A$3000 for a 20% to 40% actionable return rate. Cuffe et al^11^ reported similar amounts when they asked patients with advanced cancer what they were willing to pay for genomic tests to identify the likelihood of experiencing significant chemotherapy-related toxic effects. Thus, across both treatment and toxicity outcomes, patients with cancer appear to be constrained in what they are willing to pay.

Low willingness to pay may reflect an underlying perception of the low likelihood of extended benefit from any treatment, tailored or not (and therefore a low benefit/cost ratio). However, we did not find associations between tipping points and knowledge, which included items evaluating understanding of the current limitations of MP. Unwillingness to pay may also reflect the lack of ability to pay. Cancer treatment is costly, and financial toxicity is a growing concern. A recent US study^29^ found that 42% of patients with cancer used all their available funds—an average of A$92 000—within 2 years of diagnosis. We did not ask patients about their income; however, socioeconomic status, based on postcode, was not associated with the tipping points. Postcode-based estimates provide only a moderate estimate of socioeconomic status according to a large, population-based study in the UK,^30^ so this variable does not conclusively rule out income barriers. We found that patients without a medical- or science-related occupation required greater benefit offered by the test to pay A$10 000 than those with a medical- or science-related occupation. However, this association likely reflected a greater understanding of and belief in science in those who worked in the field.

A recent survey reported by Chow-White and colleagues^31^ found that 61% of Canadian medical oncologists thought a major challenge to routine genomic testing was cost. Already some academics are questioning the amount of money being spent on genomic testing in the Canadian health system, given the relatively low return rates and improved outcomes.^32^ As a society, we need to continue to monitor the clinical utility of tests and the extent to which they can be implemented in the most cost-effective ways in clinical practice. Nonetheless, as 166 of our participants (22%) were prepared to pay A$10 000 regardless of actionable return rates, there is a clear need to openly discuss the option of MP with patients, if it were to become routinely available. Patients for whom uncertainty was more aversive were more willing to pay A$3000 or A$10 000 for MP at lower levels of return rate (eg, 1%), perhaps feeling that greater certainty on any front, regardless of eventual treatment benefit, was worthwhile in the context of failing standard treatment options and desperation.

### Limitations and Strengths

Limitations of this study include the hypothetical nature of the questions posed, since these patients were receiving MP as part of a genomic study at no cost. Responses to hypothetical scenarios have been criticized as not accurately reflecting what people do in real life,^30^ but these scenarios are also recognized as having important advantages as a practical and efficient way to explore complex situations.^33,34^ Our sample had relatively good performance status (almost all were Eastern Cooperative Oncology Group status 0 or 1), disallowing analysis of performance status as a potential factor associated with perceived value. However, this cohort likely represents those who would be offered MP in clinical practice.

The study strengths include the fact that these participants had diseases for which MP might be used in routine practice and were offered MP; they were provided relevant information, including the low likelihood of an actionable result being found; and they were given time to consider the pros and cons of MP before reaching a decision regarding study participation. Furthermore, we had a large cohort with statistical power to assess factors with smaller effect sizes that were potentially associated with tipping points.

## Conclusions

To our knowledge, this is the first study to quantitatively examine the value that patients with advanced cancer place on MP to guide treatment in the context of the failure of other therapies. We found that many participants were highly interested in MP, but were substantially influenced by considerations of cost. Nevertheless, some participants were prepared to pay a large amount for MP, particularly as the rate of informative results increased. This variability in response suggests that it is difficult for physicians to judge on behalf of patients what their treatment preferences will be in the context of complex trade-offs. This finding suggests that a policy of open disclosure regarding the option of MP should be fostered, but this practice is not straightforward to implement in the clinic. In our practice, we are reviewing consent processes to ensure that information is available and tailored to patient need to ensure informed consent.

## Notes

Supplement. **eFigure.** Forest Plot Depicting Variables Associated With Tipping Points
