# Supplementary material for: Assessment of the Value of Tumor Variation Profiling Perceived by Patients With Cancer
Source: JAMA Netw Open. 2020 May 14;3(5):e204721. doi: 10.1001/jamanetworkopen.2020.4721 (PMC7225901; doi:10.1001/jamanetworkopen.2020.4721)
Supplement: Supplement. — eFigure. Forest Plot Depicting Variables Associated With Tipping Points [file jamanetwopen-3-e204721-s001.pdf]

Supplementary Online Content

Butow P, Davies G, Napier CE, et al. Assessment of the value of tumor variation profiling perceived by patients with cancer. JAMA Netw Open. 2020;3(5)e204721. doi:10.1001/jamanetworkopen.2020.4721

eFigure. Forest Plot Depicting Variables Associated With Tipping Points

This supplementary material has been provided by the authors to give readers additional information about their work.

© 2020 Butow, P, et al. *JAMA Network Open.*

eFigure. Forest Plot Depicting Variables Associated With Tipping Points

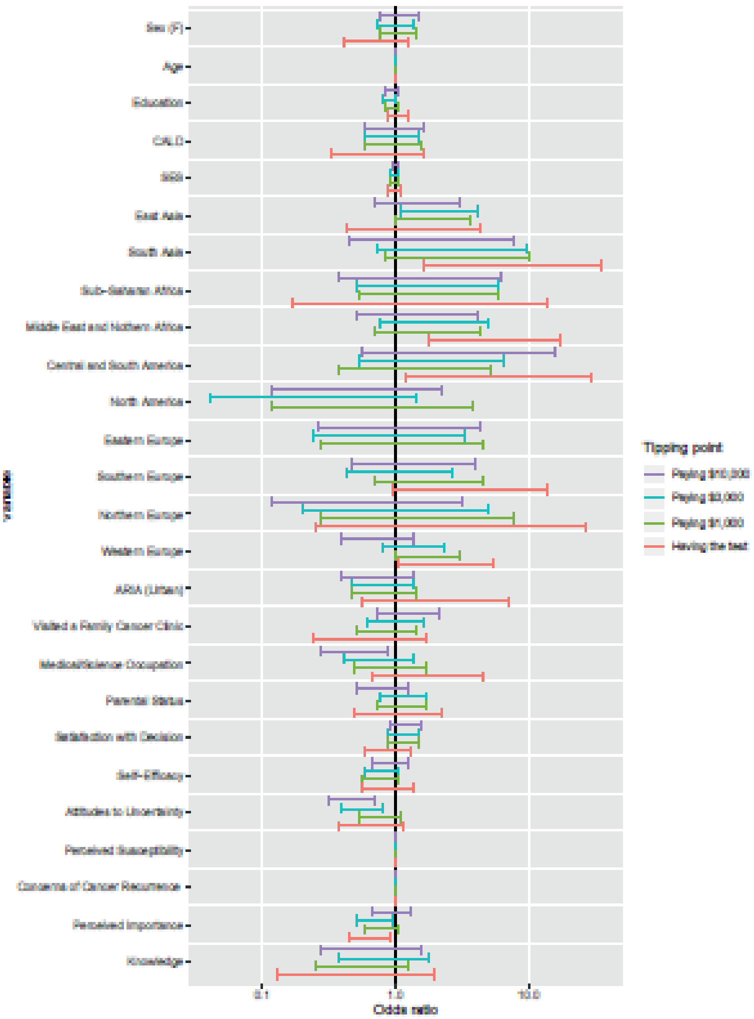

© 2020 Butow, P, et al. *JAMA Network Open.*
